# Effect of *O*. *porcinus* Tick Salivary Gland Extract on the African Swine Fever Virus Infection in Domestic Pig

**DOI:** 10.1371/journal.pone.0147869

**Published:** 2016-02-01

**Authors:** Jennifer Bernard, Evelyne Hutet, Frédéric Paboeuf, Tantely Randriamparany, Philippe Holzmuller, Renaud Lancelot, Valérie Rodrigues, Laurence Vial, Marie-Frédérique Le Potier

**Affiliations:** 1 Unité Virologie et Immunologie Porcines, Laboratoire de Ploufragan-Plouzané, Agence Nationale de Sécurité Sanitaire (Anses), Univ Bretagne Loire, Ploufragan, France; 2 Laboratoire National de Diagnostic Vétérinaire, Antananarivo, Madagascar; 3 UMR CMAEE "Contrôle des maladies animales exotiques et émergentes", Centre de coopération internationale en recherche agronomique pour le développement (CIRAD), Montpellier, France; Universidade Federal do Rio de Janeiro, BRAZIL

## Abstract

African swine fever is a haemorrhagic disease in pig production that can have disastrous financial consequences for farming. No vaccines are currently available and animal slaughtering or area zoning to restrict risk-related movements are the only effective measures to prevent the spread of the disease. *Ornithodoros* soft ticks are known to transmit the African swine fever virus (ASFV) to pigs in farms, following the natural epidemiologic cycle of the virus. Tick saliva has been shown to modulate the host physiological and immunological responses during feeding on skin, thus affecting viral infection. To better understand the interaction between soft tick, ASFV and pig at the bite location and the possible influence of tick saliva on pig infection by ASFV, salivary gland extract (SGE) of *Ornithodoros porcinus*, co-inoculated or not with ASFV, was used for intradermal auricular inoculation. Our results showed that, after the virus triggered the disease, pigs inoculated with virus and SGE presented greater hyperthermia than pigs inoculated with virus alone. The density of Langerhans cells was modulated at the tick bite or inoculation site, either through recruitment by ASFV or inhibition by SGE. Additionally, SGE and virus induced macrophage recruitment each. This effect was enhanced when they were co-inoculated. Finally, the co-inoculation of SGE and virus delayed the early local spread of virus to the first lymph node on the inoculation side. This study has shown that the effect of SGE was powerful enough to be quantified in pig both on the systemic and local immune response. We believe this model should be developed with infected tick and could improve knowledge of both tick vector competence and tick saliva immunomodulation.

## Introduction

Complex interactions have been described in many host-vector-pathogen associations. Most of these associations are highly adaptive, especially for vectors and pathogens that try to escape host defence responses by particular behavioural or immunological control strategies [1].

Ticks play an important role in pathogen transmission and emergence and are considered of great importance in veterinary and public health domains [2]. As blood-feeding parasites, ticks have to develop high plasticity and resistance to host immune systems to achieve complete engorgement. Indeed, the host reacts to the dilacerations of its own skin barrier by ticks’ mouthparts and activates an early response to infestation at the bite site [3]. This immune response involves haemostasis and inflammation modulation, as well as the development of cellular and humoral responses through the early recruitment of mononuclear phagocytic cells and polymorphonuclear cells, the maturation of antigen-presenting cells (APCs), the activation of mast cells, and other modulation of antibodies, cytokines, chemokines and the complement system [4,5]. At the same time, ticks produce pharmacologically active molecules in their saliva to escape host immune defences [6], incidentally improving pathogen transmission [7].

African swine fever (ASF) is a disease with disastrous economic consequences in pig production that can be transmitted by several soft tick species of the *Ornithodoros* genus (Acari, Argasidae). ASF is caused by a DNA virus of the *Asfarviridae* family [8], which induces lethal haemorrhagic fever in domestic pigs. Quarantine of the affected areas and the slaughtering of infected or suspicious animals are currently the only reliable control strategies [9]. ASF is highly contagious among pigs and the ASF virus (ASFV) can persist in the environment and fomites for weeks or even months. *Ornithodoros* ticks such as *Ornithodoros erraticus* in Spain and Portugal, and *O*. *moubata sensu lato* in eastern and southern Africa, are able to maintain and transmit the virus, and are competent vectors and reservoirs for ASFV [10]. These ticks can maintain ASFV for years and transmit the virus through different routes such as transovarial and/or sexual transmission from tick to tick, as well as horizontal transmission to suids via contaminated saliva or coxal fluid [11]. Given their endophilous lifestyle, *Ornithodoros* ticks are sedentary and greatly dependent on their host habitat [12].

During the early stage of pig infection with ASFV, mononuclear phagocytic cells are the main targets for viral replication [13]. At the inoculation site, ASFV induces the recruitment of macrophages, as well as their maturation, and phagocytic and secretory activation [13], through increased production of cytokines such as TNF-α, IL-1 and IL-6 [14,15]. Macrophages have essential functions in the host innate immune response and in the modulation of inflammation and maintenance of skin homeostasis [16,17]. Other important immune target cells for ASFV replication are APCs, particularly Langerhans cells (LC), which have a sentinel role in the skin. Following infection, APCs mature in interdigitating dendritic cells and present ASFV antigen in lymph nodes [18]. However, specific ASFV contamination through tick bites and the action of tick saliva on ASFV infection in pigs have never been described. Only two studies investigated immunomodulation due to *O*. *moubata* saliva [19,20] but not in relation to ASFV or other pathogen transmission. For several other tick-borne diseases, tick saliva was described as impairing the chemo-attraction or maturation of macrophages, altering the function of dendritic cells (DCs) in the transportation of nonself antigens or reducing the ability of lymphocytes to proliferate [6,21,22]. Modulation of the production of cytokines, such as TNF-α, IFN-γ or IL-10, was also reported [4,23]. In the specific case of ASFV vectorial transmission, similar patterns can be predicted with greater complexity, since these cells are also the main target of ASFV.

Since monocytes-macrophages and other APCs are recruited early when skin aggression or infection occur [5], LCs are at the first line of defence, triggering the local immune response and playing a key role in the achievement of the blood meal for ticks, and since monocytes-macrophages and APCs are the target cells for ASFV replication [13,24], we chose to focus our study on LCs and macrophage cells. The aim of this work was thus to determine whether the saliva of *O*. *porcinus* (belonging to the *O*. *moubata* complex of species) was able to modulate the immune response of domestic pigs infected by ASFV, using the intradermal injection of tick salivary gland extract (SGE) in the presence or absence of ASFV. Our observations focused both on the pig systemic immune response and on pig skin inflammation and cellular modulation (especially LCs and macrophages) at the tick bite location. Unlike previous studies, the assessment of such immune modulations was conducted on the natural hosts, domestic pigs, for soft tick vectors or ASFV and using what we believe to be a highly adapted tick-virus association with *O*. *porcinus* ticks collected from Madagascar and a Madagascan ASF virus strain.

## Materials and Methods

### ASF virus isolate

The highly pathogenic ASFV isolate Ambaton02 (GenBank accession number BankIt1774827 ANSES-MADA68322 KP144287) was originally isolated from an infected domestic pig in Ambatondrazaka, Madagascar, in 2002 and kindly provided by the Direction de la santé animale et du phytosanitaire (DSAPS)—Ministère de l'agriculture de l'élevage et de la pêche, Antananarivo, Madagascar. The Ambaton02 isolate was classified as a haemadsorbing ASFV strain belonging to the genotype II [25]. It was passaged three times in primary porcine alveolar macrophages before use in this experiment.

### Soft ticks

*Ornithodoros porcinus domesticus* ticks were collected from pig pens in Madagascar between 2006 and 2010, under the supervision of DSAPS. ASFV DNA was detected naturally in some specimens, using PCR [26]. A review of the literature indicated that this species was able to maintain and transmit ASFV [27,28]. The ticks used for this study were shown to be ASFV-free before being reared at CIRAD, Montpellier, France. They were regularly fed on artificial membrane with heparinised blood [29] and kept at 24°C, in 85% relative humidity, to complete the developmental cycle from nymph to adult stage.

For the experiments, 5 adult ticks were placed together in a Petri dish covered with a piece of mosquito netting, to allow the ticks to bite the pig through the fabric. This device was attached to the ear of a pig with adhesive tape for 2 hours to allow the ticks enough time to complete their blood meals. Another group of unengorged adult ticks was used to prepare the salivary gland extract (SGE). They were dissected and salivary glands were removed, each gland was crushed in 400 μL of Minimum Essential Medium (MEM) (BE12-611F, Ozyme, France), and the homogenates were clarified by centrifugation before inoculation. One adult tick SGE was choosen to simulate two feeding ticks.

### Pigs

Forty-eight Large-White pigs were obtained from the specific pathogen-free (SPF) breeding facilities at ANSES, Ploufragan, France. Pigs of both sexes were eight weeks old and weighed 30–35 kg at the time of inoculation. The experiment was performed in accordance with EU and French regulations on animal welfare in experimentation. The protocol was approved under number 20/12/12-15, by the French ethical committee for animal experimentation, named ComEth-Anses/ENVA/UPEC (agreement C2EA-16, Ministère de l’enseignement supérieur et de la recherche, Paris, France). The procedure for the euthanasia of the animals was based on an accepted method included in European Directive 2010/63/EU, using an anaesthetic overdose of 20 mg of sodium thiopental per kilogram of weight, administered via the vena cava. All the pigs were maintained at BSL-3 security facilities throughout the experiment and fed *ad libitum*.

### Experimental design and trial monitoring

The pigs were divided into 6 groups (Table 1). Two groups of pigs received an intradermal inoculation in one ear with virus alone (ID ASFV), or with virus and SGE (ID ASFV+SGE). In addition, four groups of pig did not receive any virus: i) one group was bitten by ASFV-free ticks (TICK), ii) one group received an intradermal inoculation of SGE (ID SGE), iii) one group received an intradermal inoculation of medium (ID MEM) and iv) one group was the negative control group (NEG).

**Table 1:** Number of pigs per trial, treatment group and time post inoculation.

Group	ID ASFV		ID ASFV+SGE		TICK	ID SGE	ID MEM	NEG
ASFV inoculation	HD trial	LD trial	HD trial	LD trial	No	No	No	No
**Nb of pig at 1hpi**	9	6	9	6	10	3	1	4
**Nb of pig at 48hpi**	6	4	6	4	8	2	0	4
**Nb of pig at 5–8 dpi**	3	2	3	2	6	0	0	4

Nb: number (of pig alive); hpi: hours post-inoculation; dpi: days post-inoculation.

Except for the NEG group, each pig received 5 intradermal inoculations of 200 μl, or 5 ticks, on one ear. The other ear was kept as an internal control to account for individual variations of cell counts (macrophages and Langerhans cells). For both the ID ASFV and ID ASFV+SGE groups, 18 pigs received 10^4^ 50% haemadsorbing doses (HAD_50_) per pig called the high ASFV dose (HD) trial and 12 pigs received 10^2^ HAD_50_/pig, called the low ASFV dose (LD) trial (Table 1).

The pigs were monitored daily as previously described [30] for rectal temperature and clinical signs (inappitence, recumbancy, skin haemorrhage, joint swelling, laboured breathing and/or coughing, ocular discharge, diarrhoea, blood in urine, vomiting), which were recorded and scored according to a scale from 0 to 5 per sign. The pigs were weighed regularly and before euthanasia. Apart from the NEG group, one to three pigs were slaughtered at 1 hour and 48 hours post inoculation (pi) in each group of pigs (Table 1). The other pigs were slaughtered between 5 and 8 days pi (dpi), as soon as the clinical score was equal or higher than 15. On post-mortem examination, gross lesions were observed and scored.

### Sample collection

Blood samples were collected before inoculation and then at least twice a week depending on the group, for several uses: (i) on heparin (Vacuette 9 ml clinical chemistry, lithium heparin Greiner Bio-One, Dutscher, France) for virus isolations, (ii) on EDTA (Vacuette 4 ml haematology, EDTA-K3 Greiner Bio-One Dutscher, France) for blood cell numbering (MS9 hematology analyzer, Melet Schloesing Laboratoires, Osny, France) and for ASFV genome real-time PCR detection.

Serum samples were purified from coagulated blood samples in dry tubes (Vacuette 8 ml Z Serum Sep Clot Activator, Greiner Bio-One, Dutscher, France) by centrifugation at 3000 g for 5 min for cytokine quantification.

Lymphoid organs (spleen, tonsils and parotid lymph nodes) were collected at necropsy for virus detection.

Both ears of each pig were collected less than 15 min after euthanasia to investigate local immune response. Two skin biopsies were taken per ear using 8 mm punches, fixed in 4% paraformaldehyde overnight, immersed in 10%, 20% and 30% sucrose baths and snap-frozen in OCT compound (MM-France, Francheville, France). Serial cryosections (12 μm) were performed using a cryomicrotome (Microm HM 505 E-VAC, Francheville, France). Slides were air-dried and labelled with anti-swine antibodies (Ab) and isotype-specific secondary antibodies and isotype control (IgG2b and IgG1, Dako, Les Ulis, France) for the immuno-histological study. Two other biopsies per pig were post-fixed in 4% paraformaldehyde overnight and embedded in paraffin according to the routine method applied at Labocea 22 (Ploufragan, France) for histological lesion analysis by a pathologist.

### Virus detection

Virus isolation on a heparin blood sample was performed as described in the OIE diagnostic manual [31]. The absence of any antagonist effect of SGE on the virus isolation was verified on pig alveolar macrophages (data not shown). Virus detection was carried out by real-time PCR as previously described [32] on EDTA blood samples and organs, after DNA extraction with the DNeasy Blood and Tissue kit (Qiagen, Courtaboeuf, France). For each pig euthanized at 48 hpi, four separate samples used for DNA extraction from the parotid lymph node on the side of the inoculated ear (PLI). A sample of spleen, tonsils, and two different samples of parotid lymph nodes from the opposite side (PLO) were extracted. Organs that were late positive for ASFV by real-time PCR (***C***_***t***_ > 40) were further analysed by a haemadsorption assay (HAD) using SPF primary porcine alveolar macrophages in a 96-well plate [31].

Viral genome loads, quantified by the cycle number threshold (*C*_*t*_) detected by real-time PCR, were categorized as i) negative (*C*_*t*_ > 45), ii) weakly positive, close to the real-time PCR detection threshold (37 < *C*_*t*_ ≤ 44), iii) positive (26 < *C*_*t*_ ≤ 36) and iv) strongly positive (15 < *C*_*t*_ ≤ 25).

### Porcine cytokine quantification

In pig sera, IL-6, IL-12 and TNF-α were measured using ready-to-use ELISA kits (R&D systems, Minneapolis, USA) and haptoglobin quantification was carried out with the TRIDELTA Development LTD kit, (Eurobio, Courtaboeuf, France). IFN-α was quantified by a homemade ELISA test as previously described [33].

### Immunofluorescence (IF) and histological staining on skin biopsies

For IF, non-specific binding of antibodies (Ab) to tissue sections was blocked for 1 h in PBS containing 3% goat serum albumin (GSA). Slides were incubated overnight with primary monoclonal antibody (mAb) in PBS containing 10% GSA and 0.03% Triton X100, then washed in PBS baths and incubated for 2 h with secondary Abs (Alexa Fluor^®^ goat anti-mouse, Invitrogen) at room temperature. Finally, Hoechst stain solution (Sigma-Aldrich, St Louis, USA) was added to the slides after PBS washes. Sections were mounted in Mowiol 4–88 medium (Sigma-Aldrich, St Louis, USA) for analysis using a fluorescent microscope. Microscopy observations were performed with an Olympus BX41 epifluorescence microscope (Scop Pro, Itteville, France). Images were recorded on an EXI Aqua camera (QImaging, Surrey, Canada) using Image Pro-Plus software (Media Cybernetics). Twelve serial skin sections of each of the 66 biopsies were systematically examined and pictures were taken of representative sections. Ten to 25 fields were captured from comparable regions of dermis in each biopsy at the same exposure and magnification by chromatic filter for panorama reconstruction. Autofluorescence intensity was subtracted before picture analysis. Manual counting of Langerhans cells (LC) was carried out by SWC3-Ab labelling (SWC3/CD172, IgG2b, porcine pan-myeloid, Clone 74-22-15A, SouthernBiotech) and by morphological discrimination on all epidermis transects of the opposite ear and above the injured transect of the inoculated ear. LC density was expressed as LC μm^-1^. We systematically delineated rectangular counting frames of identical areas to assess the density of cells per frame. Macrophages were manually counted by co-labelling using SWC3-Ab and CD163-Ab (IgG1, Monocytes and macrophages, Clone MCA2311, AbD serotec) in 4 rectangular counting frames on the opposite ear: two in the dermis and the other two in deep dermis, and on the observed lesion area made by the needle or the tick bite for the inoculated ear (S1 Fig).

Haematoxylin-Eosin and saffron staining was used for histological lesion descriptions and scored using a semi-quantitative method according to severity and intensity of the lesions as oedema (1–3), haemorrhage (4–6), inflammation (7–9), dermitis (10–12), or necrosis (13–15). A descriptive analysis of 64 biopsies was performed on the mean total score per pig group.

According to observations of histological lesions and LC distribution along the epidermis before counting, biopsies were distributed in 5 patterns: i) “Mechanical” class, which included biopsies with epidermis disruption caused by needle introduction or tick mouthparts; this class was characterized by a total absence of LCs in the disrupted region and normal density at each extremity, ii) “Physiological” class, which showed a disappearance of LCs above the lesion with gradual reparation on both sides, iii) “Scab” class, which corresponded to phenomena of wound healing; there were no LCs under the scab and gradual reparation on both sides, iv) “Deep” class, which only concerned inoculated groups with lesions in deep dermis but a non-modified LC distribution, and v) “No-effect” class, which presented a normal distribution of LCs above the lesion in the dermis.

### Statistical Analysis

We fitted statistical models to the available data, thus utilizing most of the data and limiting the number of tests. In all the models the factors of interest were the ASFV dose (categories: no ASFV, low ASFV dose, high ASFV dose), tick saliva (categories: no saliva, tick bite, salivary gland extract) and time after inoculation (categories: 1 hpi and 48 hpi).

For the rectal temperature and clinical scores, preliminary exploratory data analyses revealed sigmoid patterns for the response, with a delay before the onset of the response, followed by a steep rise and a plateau preceding the agonic stage. To model this pattern, we used a 3-parameter nonlinear logistic model [34]. The first parameter (*Ø*_1_) was the plateau value (horizontal asymptote when time increased); the second parameter (Ø_2_) was the inflection point of the response curve; the last parameter (Ø_3_) was a shape parameter (steepness of the curve around the inflection point). As the rectal temperature showed between-pig variations (range: 39.1–39.8°C on the day of experimental infection), we modelled the temperature change from the inoculation day, rather than the actual rectal temperature.

The nonlinear logistic models were fitted using generalized least-squares (GLS), which provided maximum-likelihood (ML) estimators. During the model building stage, this property was used to compare fitted models with the Akaike information criterion (AIC) corrected for the small sample size [35,36]: AIC = −2 log(*L*) + 2 *k* and AICc = AIC + 2 *k* (*k* + 1) / (*n* − *k* − 1), where *L* was the maximized likelihood, *n* was the number of observations and *k* was the number of parameters (coefficients) in the model. AICc was used to compare models with the same response. Comparable models with lower AICc were considered as better than those with higher values.

Moreover, the GLS estimation method made it possible to account for correlations in the model residuals related to repeated measurements made on the same pigs, as well as possible heteroscedasticity in these residuals [34]. The pig was defined as a grouping factor, and a homogeneous within-pig correlation structure was used for residuals.

For the detection of ASFV DNA in parotid lymph nodes with real-time PCR, we considered the cycle number threshold (*C*_*t*_) as a quantitative variable. We were also confronted with repeated measurements in this analysis, with several values of *C*_*t*_ recorded on the same lymph node, and two parotid lymph nodes per pig. We used a two-level linear mixed-effect model [37], with ASFV dose, tick saliva and time as the fixed effects, and two nested random effects (grouping factors) associated with the intercept: (i) pig, and (ii) parotid lymph node within pig. Models were fitted with an ML method [34].

For the analysis of Langerhans cells and macrophage density, the within-pig correlation of residuals was removed, taking each pig as its own control. Indeed, for each pig *i*, we computed:

- The cell density (Langerhans cells or macrophages) in non-inoculated (control) ears: *d*_*i*,*ref*_ = Σ_*i*_ *У*_*i*,*ref*_ / Σ_*i*_ *Z*_*i*,*ref*_ where *y*_*i*,*ref*_ was the cell count in region *Z*_*i*,*ref*_. For Langerhans cells, *Z*_*i*,*ref*_ was a line transect of known length (*Z*_*i*,*ref*_) () located in the epidermis. For macrophages, it was a counting frame of known area(*Z*_*i*,*ref*_),
- The cell density in inoculated (treated) ears: *d*_*i*,*obs*_ = Σ_*i*_ *У*_*i*,*obs*_ / Σ_*i*_ *Z*_*i*,*obs*_. For Langerhans cells, *Z*_*i*,*obs*_ was a transect line segment chosen just above the inoculation point. For macrophages, the counting frame was selected close to the inoculation point in an area with a higher macrophage density as determined after a preliminary visual inspection,
- The difference in cell density *δ*_*i*_ = d_*i*,*ref*_ − d_*i*,*obs*_. A positive value of *δ*_*i*_ indicated a decrease in cell density. This difference in cell density was the response in subsequent statistical models.

We used linear models with adaptations required by response peculiarities. Indeed, *δ*_*i*_ was a difference of cell counts which typically showed a high range of values, with outliers (observations very different from the population mean). To account for this, we used a robust linear model, in which outliers had a limited influence on coefficient estimates [38].

Model coefficients were fitted by iterated re-weighted least squares. Moreover, because of the small sample size (24 pigs allocated to different treatment categories), it was difficult to use the asymptotic condition for the computation of *p* values associated with tests on model coefficients. Instead, we used a bootstrap procedure on model residuals as described by Davison and Hinkley [39]. In short, the estimated coefficients (robust linear model) and fitted values were considered as fixed values. Residuals were sampled with replacement, and a new response was computed adding the fitted values to the sampled residuals. The robust linear model was then refitted with this new response, and the resulting coefficients were stored. This loop was iterated *B* times (with large values of *B*: typically 999 or 1,999, or more). The *B* sets of coefficients were added to the original set. The 2.5% and 97.5% quantiles of each coefficient series of *B*+1 values (or linear combination of these coefficients) were then used as 95% confidence intervals. For instance, the fitted mean difference in cell density ${\hat{\bar{\delta }}}_{i}$ was a linear combination of model coefficients. To know whether the combined effects of time, ASFV dose and tick saliva (including possible interactions) significantly altered ${\hat{\bar{\delta }}}_{i}$ (null hypothesis: ${\hat{\bar{\delta }}}_{i}\;=\;0$), we computed the *B*+1 values for a given group (e.g. tick saliva and high ASFV dose at 48 hpi), and assessed whether the 95% confidence interval included the value of 0 (non-significant combined effect for α = 0.05) or not (significant combined effect for α = 0.05).

## Results

### General pig immune response: clinical signs and pathophysiology

#### 1. Clinical scores and viraemia

Symptoms were only observed when the virus was inoculated, with no clinical signs for the NEG, TICK and ID SGE groups throughout the experiment. The disease spread and clinical scores were similar between intradermally infected groups whatever the virus doses (HD and LD trials), including first a loss of appetite at 1–3 dpi, then hyperthermia the following day, at 2–4 dpi. Pigs that received the high ASFV dose were all euthanized 5 or 6 dpi for ethical reasons, whereas the pigs inoculated with the lowest dose had to be euthanized at 8 dpi. The onset of the disease, according to the clinical scores, was significantly delayed by one day in the LD trial compared to the HD trial (*p* = 5 10^−3^). The mean daily weight gain (MDWG) was evaluated and all the infected groups (ID ASFV, ID ASFV+SGE) lost weight from 3 dpi whatever the virus doses expressed in mean ± standard deviation (SD), -0.9 ± 0.1 (n = 5) kg/day for the HD trial and -0.7 ± 0.1 (n = 4) kg/day for the LD trial, whereas the non-infected groups (NEG, TICK, ID SGE, ID MEM) gained weight up to 8 dpi (0.9 ± 0.1 (n = 7) kg/day).

**Fig 1:**
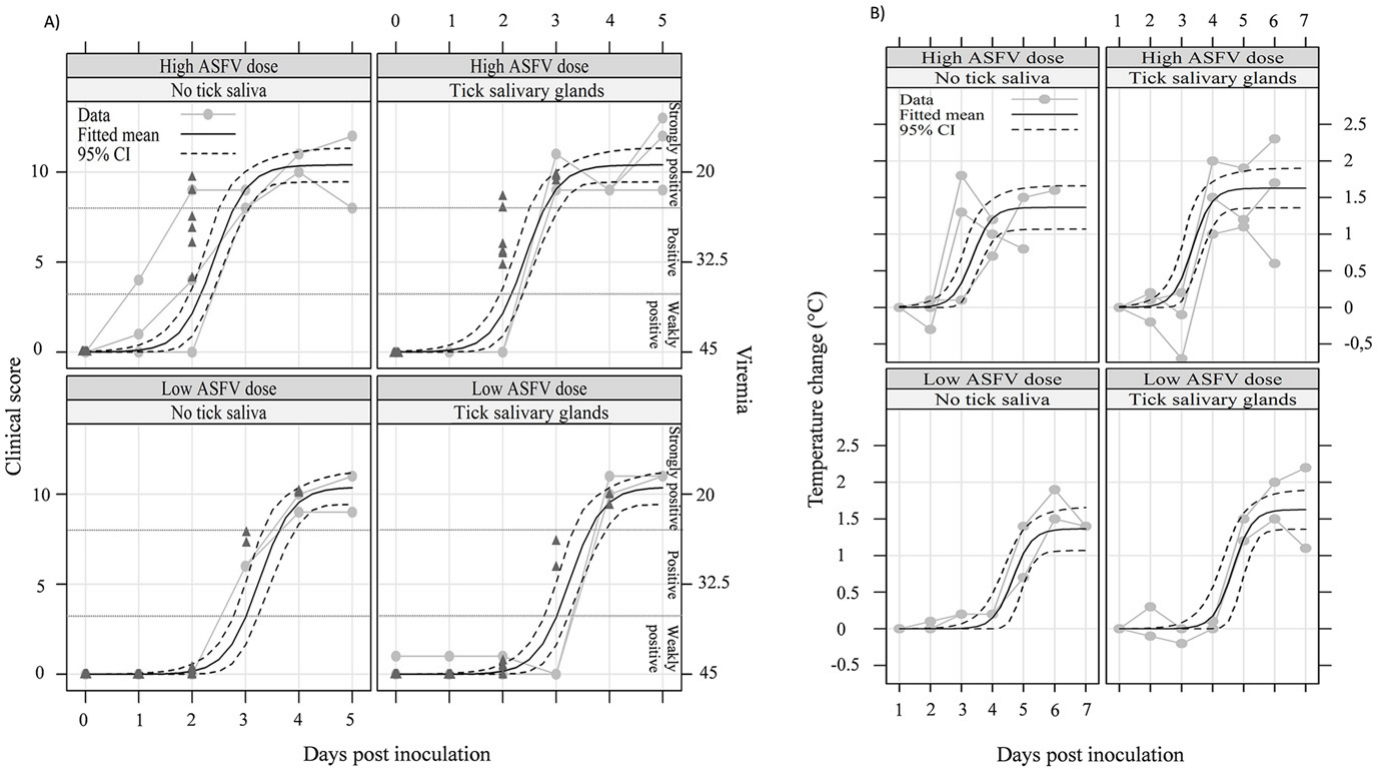
Clinical scores, viraemia and rectal temperature. (A) The scale for clinical score is shown on the left, and the scale for individual real-time PCR results is shown on the right. Real-time PCR results (dark grey triangles) were categorized as negative (*C*_*t*_ > 45), weakly positive (44 ≥ *C*_*t*_ > 37), positive (36 ≥ *C*_*t*_ > 26) and strongly positive (*C*_*t*_ < 25). (B) Changes in pig rectal temperature were recorded with respect to the inoculation day. The mean was estimated with a nonlinear logistic model fitted by generalized least squares.

The body temperature indicated that the pigs of the non-infected groups never displayed hyperthermia (T>40°C). For the other groups, the onset of fever occurred with a significant one day delay between pigs of the HD trial at 3.3 dpi and the LD trial at 4.6 dpi (*p* < 10^−3^, nonlinear logistic model) (Fig 1B). In the HD and LD trials, higher mean rectal temperatures were observed for the ID ASFV+SGE groups over the first three days after the onset of hyperthermia. To assess this effect, we considered both groups together and analysed the temperature data on the first three days after the onset of hyperthermia. We fitted the mean temperatures with a generalized least squares linear regression model with a single fixed effect (tick saliva vs. no tick saliva) and pig as the grouping factor (within-pig homogeneous correlation structure). The fitted mean temperature of the ID ASFV+SGE group was significantly higher by 0.3°C (*p* = 0.032) than for the ID ASFV group.

The virus was detected in blood as early as 2 dpi whatever the virus dose. The virus load was scored as “positive” to “strongly positive” for the 12 pigs slaughtered at that time in the HD trial, but “weakly positive” for 5 out of the corresponding 8 pigs in the LD trial and “negative” for the last 3 pigs. This difference in detection level between trials was also found at 3 dpi. Then, from 4 dpi to the endpoint, no difference was found between groups, with or without SGE, in both trials (Fig 1A, left scale).

#### 2. Blood leukocyte count

Peripheral white blood cell counts for lymphocytes, monocytes and granulocytes were similar between pig groups before virus inoculation. Leucopoenia was observed at the onset of hyperthermia in all groups of infected pigs and showed a similar pattern with a recovery period during the hyperthermia phase (Table 2).

**Table 2:** Blood leucocyte mean count kinetics (x 10^6^ cells/ml of blood) presented in relation to hyperthermia.

Group	ID ASFV		ID ASFV+SGE		ID SGE*
ASFV inoculation	HD trial	LD trial	HD trial	LD trial	No
**Leucocyte count before hyperthermia**^1^	7.4 ± 2.2	10.7 ± 2.3	9.2 ± 0.7	8.3 ± 2.4	11.1 ± 5.6
**Leucocyte count the day of hyperthermia**^2^	7.9 ± 2.1	5.4 ± 0.7	4.8 ± 1.5	5.5 ± 0.2	11.7 ± 4.7
**Leucocyte count after hyperthermia**^3^	17.4 ± 5.6	13 ± 7.9	16.1 ± 6.6	13.8 ± 7	14.9 ± 5.5

Data in x 10^6^ cells/ml of blood, Mean ± SD;

^1^: Incubation time before the onset of hyperthermia;

^2^: day of the onset of hyperthermia;

^3^: time after the onset of hyperthermia up to the end of the experiment,

*ID SGE group did not display hyperthermia, so results were separated in 3 periods of 2 days from 0 dpi to 6 dpi.

#### 3. Gross pathological lesions

Post-mortem examination did not reveal any difference between ASFV infected groups, with jaundice and splenomegaly systematically observed at 5–8 dpi. The most recurrent lesions for 15/20 pigs were severely congested or haemorrhagic lymph nodes. When pigs were slaughtered at 1 hpi, no lesions were recorded. A slight difference was observed at 48 hpi in the HD trial, with less reactive lymph nodes for the ID ASFV group than for ID ASFV+SGE, which also showed petechial lesions on the thymus. This distinction was not found for the LD trial.

#### 4. Cytokine quantification in serum

Due to technical constraints, blood samples were only taken daily for the pigs receiving the low ASFV dose (with or without SGE). For these animals, the response pattern was similar for IL-12, IFN-α and TNF-α (S2 Fig, columns 2 and 4): a rise in cytokines was observed at 4 dpi. Because of the sampling framework, we could only assess the effect of the ASFV dose for animals receiving SGE (S2 Fig, columns 3 and 4). At 3 dpi, an increase in the TNF-α level was found in the HD trial, whereas it was significantly delayed by one day in the LD trial (*p* = 0.02, Wilcoxon test). The same pattern was observed for IFN-α, IL-12, and I-L6. A rise was also observed for haptoglobin (data not shown) but it remained under the positivity threshold recommended by the manufacturer for detecting inflammation.

#### 5. Virus detection in organs

**Fig 2:**
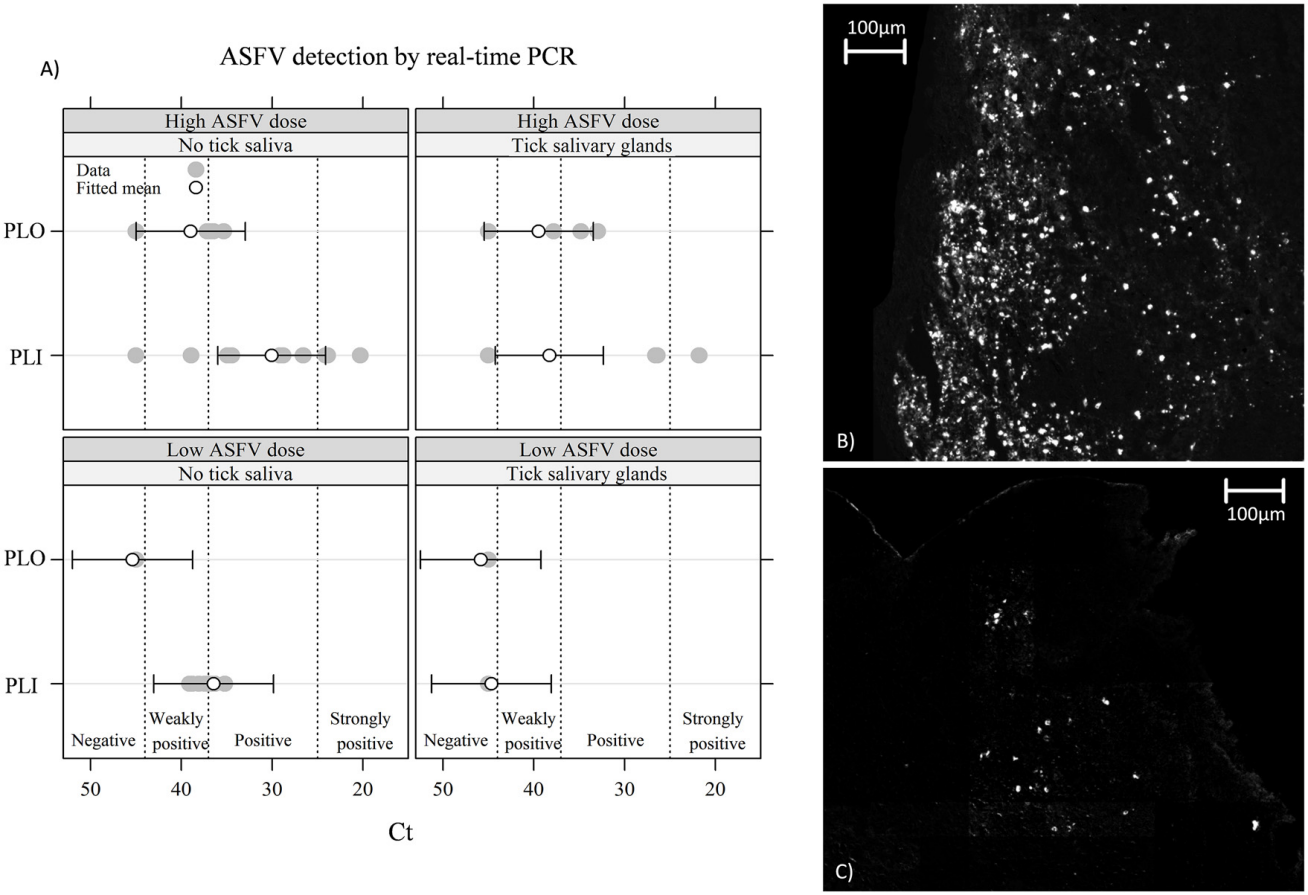
Viral detection in parotid lymph nodes at 48 h pi. (A) Quantification of ASFV DNA using real-time PCR. Real-time PCR results were categorized as negative (*C*_*t*_> 45), weakly positive (44 ≥ *C*_*t*_ > 37), positive (36 ≥ *C*_*t*_ > 26) and strongly positive (*C*_*t*_ < 25). The mean was fitted with a linear mixed-effect model. (B) Virus labelling at 48 h pi in parotid lymph nodes (inoculation side) for pigs receiving a high ASFV dose alone. (C) Virus labelling at 48 h pi in parotid lymph nodes (inoculation side) for pigs receiving a high ASFV dose and tick salivary gland extract. Virus protein VP72 was labelled by immunofluorescence. PLI: parotid lymph node on inoculation side; PLO: parotid lymph node on opposite side.

At 1 hpi, no viral genome was detected in any lymphoid organ. Conversely, all pigs euthanized at 5–8 dpi presented a very high level of viral genome in their lymphoid organs. The results were more heterogeneous when pigs were euthanized at 48 hpi. In parotid lymph nodes (Fig 2), the viral load was higher in the high ASFV dose trial than in the low ASFV dose trial, for each side (inoculated or opposite side). However, this effect was not significant (*t* test, df = 7, *t* = -1.90, *p* = 0.10).

For opposite parotid lymph nodes (PLO), no difference was observed between the ID ASFV group and the ID ASFV+SGE group (Fig 2A, Wald test, *w* = 0.016, df = 1, *p* = 0.90). For inoculated parotid lymph nodes (PLIs), SGE had a large and significant effect (Fig 2A, Wald test: *w* = 4.6, df = 1, *p* = 0.031). The IF method on lymph node cryosections confirmed that PLIs of the ID ASFV group presented more labelled cells for the ASFV VP72 virus capsid protein than the ID ASFV+SGE group on a similar area of lymph node section (Fig 2B and 2C).

For the spleen and tonsils, no effect of SGE was observed (data not shown).

### Local immune response of pigs: lesions and cell recruitment in auricular skin

#### 1. Auricular biopsy lesions

One hour pi, macroscopic lesions were greater for the TICK group than for the inoculated groups, with a well characterized inflammation ring of 0.8 ± 0.2 (*n* = 19) and 0.4 ± 0.1 (*n* = 25) mm in diameter (mean ± SD) (*t* test, df = 23.5, *t* = 6.6, *p* = 9 10^−7^). This difference was more striking at 48 hpi, 1.9 ± 0.5 (*n* = 19) and 0.4 ± 0.1 (*n* = 28) mm in diameter (*t* test, df = 19.5, *t* = 11.9, *p* = 2 x 10^−10^) (S3 Fig). At the endpoint of the experiment, no more macroscopic lesions were observed except for the ID ASFV and ID ASFV+SGE groups, which only displayed yellowish pigmentation of the skin.

This observation was partially confirmed by lesion scoring. At 1 h pi, the lesion scoring by groups was slightly different, with a mean value of 17.1 for the TICK group compared to 13.3 for the ID ASFV group, 10.0 for the ID ASFV+SGE group and 6.5 for the ID SGE group (Kruskal-Wallis χ² = 7.3, df = 3, *p* = 0.06). At 48 hpi, the mean value of lesion scoring was 21.7 for the TICK group and 21.6 for the ID ASFV+SGE group, 13.8 for the ID ASFV group, and 11.0 for the ID SGE group, but there was no significant difference between groups (Kruskal-Wallis χ² = 3.8, df = 3, *p* = 0.28). From 5 dpi to 8 dpi, lesion scoring was 22.8 for the ID ASFV group and 17.3 for the ID AFSV+SGE group, while it declined to 12.0 for the TICK group (no result for the ID SGE group) and displayed an almost significant difference (Kruskal-Wallis χ² = 5.4, df = 2, *p* = 0.06).

#### 2. Langerhans cell density

**Fig 3:**
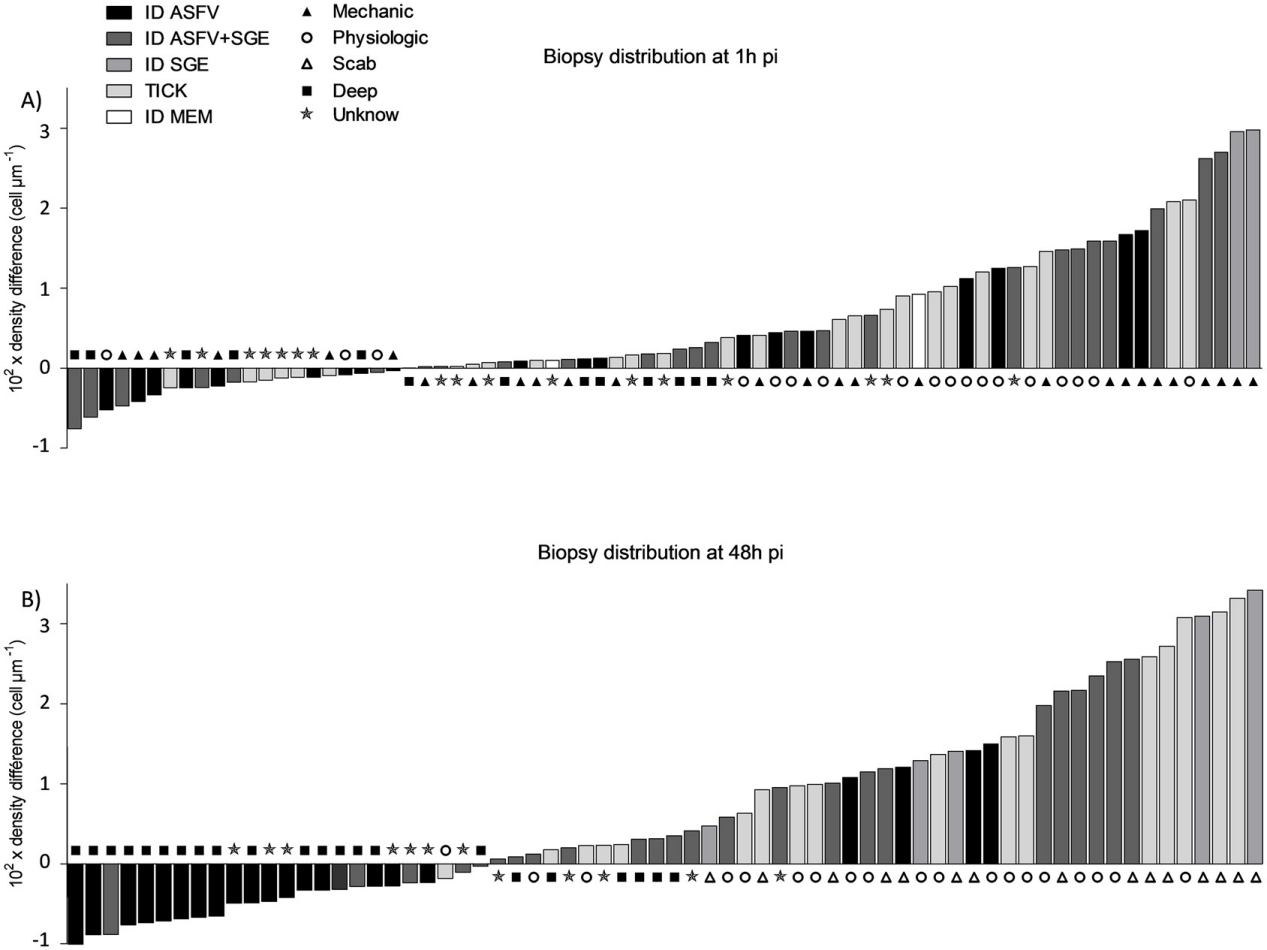
Classification of biopsies by Langerhans cell density differences and associated histological lesion pattern. (A) Classification of biopsy at 1 h pi. (B) Classification of biopsy at 48 h pi. Results of manual count and observation carried out on each slide. Two main lesion patterns were observed, one due to the tick bite or to the intradermal inoculation (pattern: mechanical or deep) and the other due to the physiological effect of saliva, SGE and virus (pattern: no-effect, physiological or scab).

**Fig 4:**
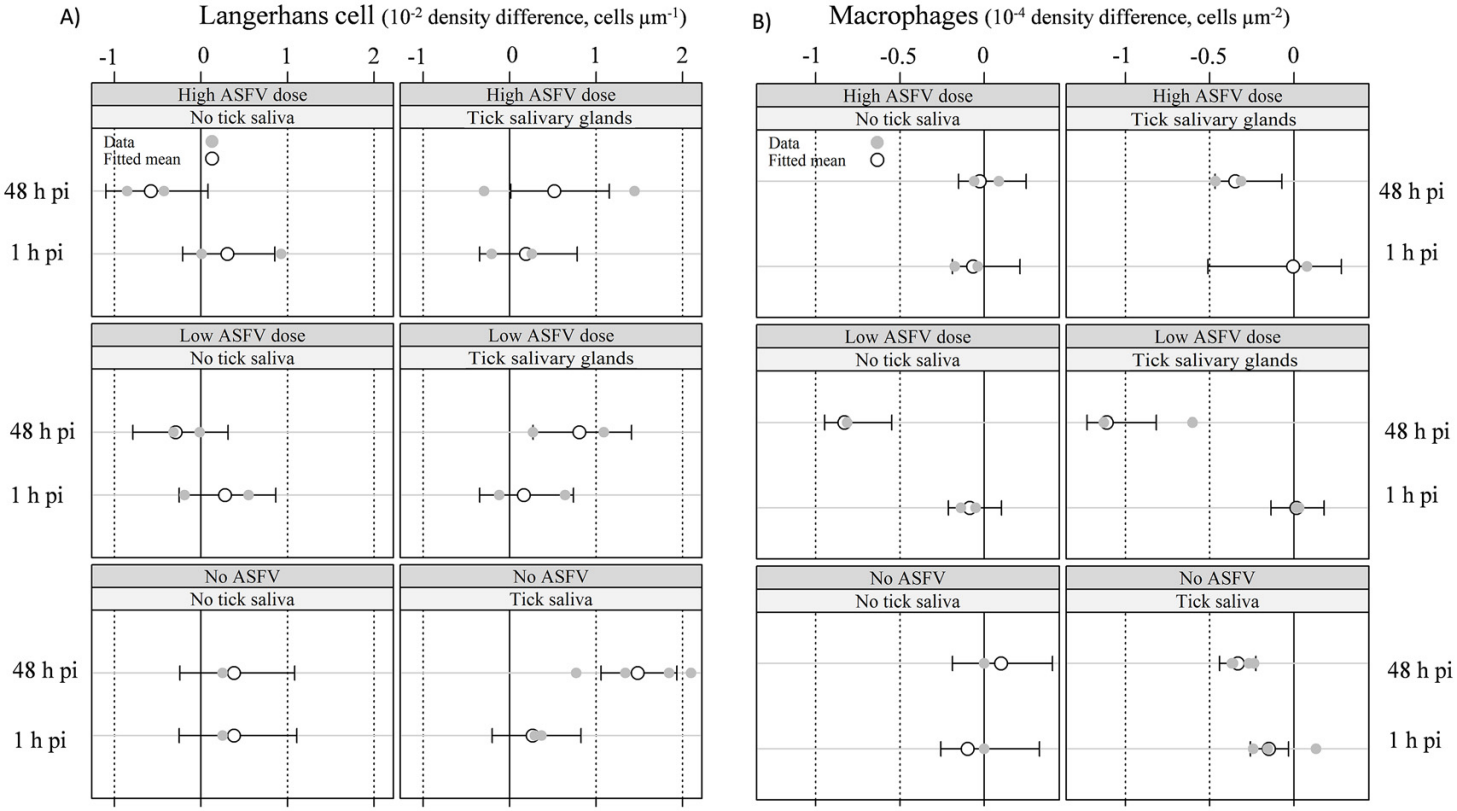
Pattern of immunological cell density difference between inoculated ear and opposite ear. (A) Langerhans cell density difference 10^−2^ x density difference, μm^-1^. (B) Macrophage density difference 10^−4^ x density difference, μm^-2^. The mean was fitted with a robust linear model.

Preliminary investigations revealed no difference in Langerhans cell (LC) density between tick bites or SGE inoculation. These two groups of pigs were therefore grouped together in the tick saliva category. The main results are shown in Figs 3 and 4A.

At 1 hpi, changes in LC density were related to all types of epidermis disruption except scabs (Fig 3A), independently of ASFV or tick saliva conditions. Indeed, no significant difference in LC density was detected between the pigs in any group: Fig 4A, second row of each group, Monte Carlo simulation, *B* = 1,999, *p* > 0.05.

At 48 hpi, most of the slices showing a decrease in LC density were from animals that received tick saliva (ID ASFV+SGE, ID SGE, TICK): Fig 3B, right-hand side. Those with the largest decrease (32/44) were categorized in the “Scab” or “Physiological” classes. Slices showing an increase had mostly been taken from pigs receiving ASFV only (Fig 3B, left-hand side). Most of them were categorized in the “Deep” class (16/24). The results from the robust linear model showed significant interaction between tick saliva and time (48 hpi): Monte Carlo simulation, *B* = 1,999, *p* = 0.006. As shown in Fig 4A, saliva significantly decreased LC density for any ASFV dose (including no virus): right-hand column, first row of each group, Monte Carlo simulation, *B* = 1,999, *p*<0.05. For pigs which did not receive tick saliva (Fig 4A, left-hand column, first row of each group), the presence of ASFV tended to increase LC density, with a higher density for the higher ASFV dose. However, this effect was not significant (Monte Carlo simulation, *B* = 1,999, *p* > 0.05).

#### 3. Macrophage recruitment in the dermis

An example of histological observations is shown in S4 Fig. As for LC, observations from tick bites and SGE inoculations were grouped for data analysis. The estimated macrophage density (robust linear model) did not show cell recruitment at 1 hpi in any pig groups (Monte Carlo simulation, B = 1,999, *p* > 0.05), with the exception of pigs receiving tick saliva alone (Fig 4B). At 48 hpi, the results showed a strong and significant interaction between the low ASFV dose and time (Monte Carlo simulation, B = 1,999, *p* = 0.01). In addition, the interaction between saliva and time was significant (Monte Carlo simulation, *B* = 1,999, *p* = 0.03). All pig groups receiving ASFV and/or tick saliva showed significant macrophage recruitment, except the pig group treated with a high ASFV dose without tick saliva (Fig 4B, left-hand column, and first row of upper group: Monte Carlo simulation, *B* = 1,999, *p* > 0.05). The pig group that did not receive either ASFV or tick saliva did not show any macrophage recruitment (Fig 4B, left-hand column, and first row of lower group: Monte Carlo simulation, *B* = 1,999, *p* > 0.05).

## Discussion

The role of *Ornithodoros* ticks in the ASF epidemiological cycle is fairly well understood, whereas the effect of tick bites, infected or not, on the immune response to infection in domestic pigs has never been studied. To recreate as closely as possible natural conditions of contamination by tick bites, we selected a combination of ticks and viral isolate collected from a restricted geographical zone (Madagascar), along with intradermal administration of a low virus dose presumed to better mimic the quantity of virus inoculated by ticks during their blood meal [27]. Extracts of *O*. *porcinus* salivary glands were used for intradermal inoculation, as it has been previously showed that there was a good correlation between transcript and protein abundance in salivary gland and saliva [40]. This was confirmed by our experimental results, with similar LC and macrophage recruitment patterns between the SGE and TICK groups.

The disease course observed in pigs, was similar to that previously described in infection by the ASF virus using other inoculation methods (intramuscular versus oro-nasal) [41–43]. This confirmed that the Madagascan ASFV strain therefore displayed a similar infection pattern to that described with the Georgian strain, which is phylogenetically very close [25,44].

### Effect of virus dose on the kinetics of immunological and symptomatic responses in pigs

Our results showed a correlation between the dose of virus inoculated (LD/HD trials) and the time course of the effects observed, with a delay of one day in the immune response, onset of clinical signs and virus dissemination in pig at 48 hpi, as previously described [43,45]. The other result attributable to the virus dose was a difference in macrophage density detected in the skin biopsies at the inoculation point at 48 hpi. It has been shown that the virus is capable, via the caspase-3 protein, of triggering the late activation of apoptosis between 24 and 48 h after infection, enabling massive dissemination [46,47]. It may be that the biopsies analysed at 48 hpi were taken at the time of this first and intense phase of virus dissemination, in which case the local disappearance of macrophages was probably due to induced cell death. A second hypothesis concerns the kinetics of macrophage maturation: once stimulated by its interaction with a pathogen, the macrophage undergoes profound changes [46,48]. SWC3/CD172-Ab, a porcine myelomonocytic marker used, in our study, to detect macrophages [18,49], is a homologue of the epitope SIRP-α [50], whose expression is regulated according to macrophage maturation [51,52]. A high virus dose can influence LC and macrophage maturation at 48 hpi [53] and induce an extinction of the SIPR-α signal, possibly reducing the detection of these cells in our samples.

### The presence of SGE modulates the immune response in pigs

#### 1. SGE plus ASFV increased fever

The group of pigs receiving an intradermal inoculation of SGE without virus displayed similar results to those from the TICK group, whether for immune or physiological responses. When the pigs were inoculated with the virus, irrespective of the dose, SGE presence increased the degree of hyperthermia, whereas SGE did not affect the systemic level of the proinflammatory cytokines TNF-α and IL-6. The pyrogenic substance hypothesis did not therefore appear to explain this difference, but it needs to be investigated further, even though very little is currently known about fever-triggering mechanisms [5].

#### 2. SGE had an immunomodulating effect on skin tissue lesions

During engorgement, the tick maintains a passageway to the outside via its saliva until 60 min [12], which is of greater consequence than a needle-mediated intradermal inoculation of a few seconds. This difference partly explains the results of the lesion observations at 1 hpi, for which the TICK group had a higher lesion score than the ID SGE group, a trend that was confirmed at 48 hpi. For inoculation with a virus+SGE mixture, the lesion score at 48 hpi then became equivalent to that of the TICK group, thereby showing a greater immunomodulating effect of SGE in the presence of virus. This mechanism should probably be considered jointly with the observation of greater hyperthermia in the groups of diseased pigs with SGE. To assess the scores of the biopsies at the end of the experiment (5 to 8 d pi), it should be remembered that the main symptom of the ASF virus is rapid-onset haemorrhagic fever. Thus, the higher lesion level in the ID ASFV group than in the ID ASFV+SGE group can be explained by the physiological consequences due to the fast multiplication of the virus. However, most saliva molecules enable ticks to modulate the haemostasis of their host [54] along with the associated scarring phenomena [6].

#### 3. SGE promoted LC disappearance in the epidermis

It has been established, that LCs are recruited by the ASF virus [18,24] and their migration may depend on the production of TNF-α induced by macrophages and neutrophils [55]. Inversely, the immunomodulation by saliva at 48 hpi was reflected in a clear reduction in the number of LCs. It is quite possible that exposing immature DCs to tick saliva leads to reduced migration and poor LC renewal [56]. In addition, LCs are sentinels with a limited radius of action from the epidermis, which are rapidly replaced by dendritic cells and macrophages in the case of deeper lesions of the dermis. The reduction observed in the ID ASFV+SGE groups was lower than for the TICK group but remained statistically similar, meaning that the salivary gland extract had an inhibiting impact on LC density, whereas it was inoculated more deeply than saliva during natural tick engorgement.

#### 4. SGE promoted the recruitment of macrophages in the dermis

Once the epidermis and the LC barrier have been overcome, pathogens encounter a second line of defence mainly consisting of a massive arrival of immune cells (e.g. neutrophils, granulocytes then macrophages) [6,17,57,58,59] However, the cell type involved early in the host-tick response also depends on the time since engorgement and the degree of host susceptibility [58,59]. The activity of macrophages would appear to be inhibited in susceptible vertebrate hosts during engorgement by hard ticks or SGE inoculation [22,60,61]. Only one study on the evasion strategy of *Dermacentor variabilis* shows substantial macrophage recruitment [62]. Our results showed weak macrophage recruitment at 1 hpi whichever group of pigs was studied, that was probably the direct consequence of the lesions caused by inoculation or tick engorgement [17]. Nevertheless, it clearly intensified at 48 hpi, that mean SGE and virus displayed macrophage recruitment alone or co-inoculated. The nature of activation signals received by these macrophages, being probably different in maturation or susceptibility to infection, could explain the differences observed between groups.

#### 5. SGE delayed virus infection in the first lymph node

Once the virus enters the host organism, it spreads rapidly by way of infected antigen-presenting cells which travel via the lymph or blood towards target organs [33,62,63]. Our results indicated that dissemination of the ASFV was found to be less in the PLIs of the pigs for which the inoculum contained SGE, suggesting that saliva molecules act more on modulating APCs. Three hypotheses can be put forward to explain this early local phenomenon *in vivo*. The first is based on the idea of dilution [64], the share of APCs bearing antigens of the virus would appear to be reduced to the benefit of those bearing SGE antigens [65–67]. Marquet et al. [64] estimated that the number of LCs migrating to the lymph nodes via the lymph amounted to around 11% of the afferent DCs. It is known that APCs and especially LCs are able to present antigens of hard tick SGE to T lymphocytes in lymph nodes *in vitro* and *in vivo* [65–67]. The second more likely hypothesis would be that SGE acts on APCs via immunoregulation phenomena. Saliva molecules might induce APC cell death or inhibit their migration to the PLIs, making virus replication less efficient in the latter. However, APCs density in the lymph nodes was similar in presence or absence of SGE (S5 Fig). The third hypothesis, which does not exclude the second, depends on certain *in vitro* and *in vivo* studies showing that hard tick saliva is capable of downregulating APC functions [23,56,68]. For instance, SGE of *O*. *porcinus* might influence the ability of APCs to present viral antigens to T cells in lymph nodes.

### Limitations and prospects

One aspect to consider in histological studies is the type of syringe administration. As we saw in our study, the inoculation depth and the concentrations inoculated are relatively dissimilar to those of tick bites [3]. In addition, the virus produced on pig macrophages did not suffer the selection pressures that its passage through the different tick tissue barriers might entail, particularly the midgut and salivary glands. However, despite these conditions, we were able to see that the effect of the virus on one hand, and the effect of SGE on the other, were strong enough to be observable and quantifiable at the inoculation point on histological sections, and physiologically in pigs. Another bias to consider was the SGE utilisation because the profile of secreted proteins in saliva is not exactly the same. However our experimental results and statistical analysis, notably on the comparison between TICK and ID SGE group, showed that it was possible to assimilate SGE effect to saliva effect.

However, the observed immune modulations still depend on the models studied and the arthropod-pathogen-host combinations concerned [69]. Barratt-Due et al. [70] showed that the OmCI molecule present in *O*. *moubata* saliva is able to modulate haemostasis in pigs *in vivo*, attenuate the production of TNF-α and IL-6, and reduce neutrophil migration, whereas their previous study had shown that TNF-α was not modulated by this protein *in vitro* in pig cells [71]. Even though some common patterns seem to occur, relationships can be difficult to model, making predictions about reactions problematical. Indeed, most vectors modulate the immune system of the host, thus facilitating pathogen proliferation. Our study showed that the molecules contained in SGE may have partly caused an inhibiting effect on macrophage and APC activation. However, we saw that SGE and tick saliva increased macrophage recruitment in the dermis, which is likely to promote viral infection. Consequently, to better understand the local effect of saliva, it would be interesting to investigate, as a priority, how the bite of a tick infected with the ASFV affects modulation of the immune cells. Further research comparing expression levels for some cytokines of interest (TNF-α, IL-6, etc.) and their tissue location (dermis and lymph node), as well as the dissemination of SGE molecules in lesions, would help to verify the hypotheses put forward. Another valuable aim of further study would be to acquire the ability to keep track of changes in LCs and antigen-presenting cells. It would thus be possible to improve our knowledge of the impact of SGE on macrophage recruitment and APC exchanges with lymph nodes. Lastly, it seems important to check that the infection patterns produced are similar between the different *Ornithodoros* species of interest (*O*. *moubata* and *O*. *erraticus* in particular). The *O*. *erraticus* tick is known to cause greater inflammation and lesions in tissues than a bite from ticks of the *O*. *moubata*–*O*. *porcinus* complex [72]. Such a study might help to more effectively characterize immunomodulation phenomena within the same tick genus (e.g. *Ornithodoros*) in line with their vector ability.

## Supporting Information

S1 Fig Cell count method on skin biopsies from inoculated ears. Within the epidermis, the transect for the Langerhans cell count is delineated in blue. Within the dermis and deep dermis, the macrophages were counted inside the areas delineated in yellow. The circular area corresponds to the area of the tick bite or inoculation point. The yellow rectangles are outside the area where the tissue was disrupted. In the opposite ear, macrophages were only counted in the yellow rectangles as there was no tissue disruption and the transect extended throughout the biopsy.
(TIF)

S2 Fig Cytokine response: dosage of TNF-α, INF-α, IL-6 and IL-12 in pig sera. For each cytokine, whatever the pig group and day post infection, the results were transformed into a percentage of serum concentration, with the maximal observed concentration considered to have 100% activity.
(TIF)

S3 Fig Histological slice of *O*. *porcinus* tick bite. (A) Tick bite at 1 h pi, black star showing the tick bite, an arrow indicating the haemorrhagic area. (B) Tick bite 48 h pi with arrows showing extensive haemorrhagic area and dermis lesions. Staining: Hemacolor kit (Merck Millipore, Darmstadt, Germany)
(TIF)

S4 Fig Effect of a tick bite and intradermal inoculation at 1 h pi in pig skin with SWC3/SC172 cell labelling. (A) Tick bite. (B) Intradermal inoculation. Two types of tissue lesion were observed. The first was characterized by an area delimited by tissue disruption, collagen disruption and more abundant SWC3/CD172 cell labelling than in healthy tissue (yellow arrows). The second was sometimes observed inside the first, consisting of more intensive damage with more abundant haemorrhage and SWC3/CD172 cell labelling (red arrows). Most of the TICK biopsies presented both areas. However, statistical model analyses were only performed on the disrupted area indicated in the photo by the yellow arrows. Biopsies in the samples with tick bites were performed to a depth of 550,0 μm (n = 14 biopsies on 7 pigs), unlike the inoculated biopsies which were performed to a depth of 1052.8 ± 511.4 μm (n = 23 biopsies on 14 pigs).
(TIF)

S5 Fig Histological slice of parotid lymph node, inoculation side, at 48 h pi. (A) Parotid lymph node from inoculation side for pigs receiving a high ASFV dose and tick salivary gland extract. (B) Parotid lymph node from inoculation side for pigs receiving a high ASFV dose alone. Merging of histological slice labelled in red with S100-Ab (IgG1, interdigitating dendritic cells, Clone SH-B1, Sigma) [18] and in green with SWC3-Ab.
(TIF)
