# Supplementary figures and images for: Effect of O. porcinus Tick Salivary Gland Extract on the African Swine Fever Virus Infection in Domestic Pig
Source: PLoS One. 2016 Feb 1;11(2):e0147869. doi: 10.1371/journal.pone.0147869 (PMC4734713; doi:10.1371/journal.pone.0147869)

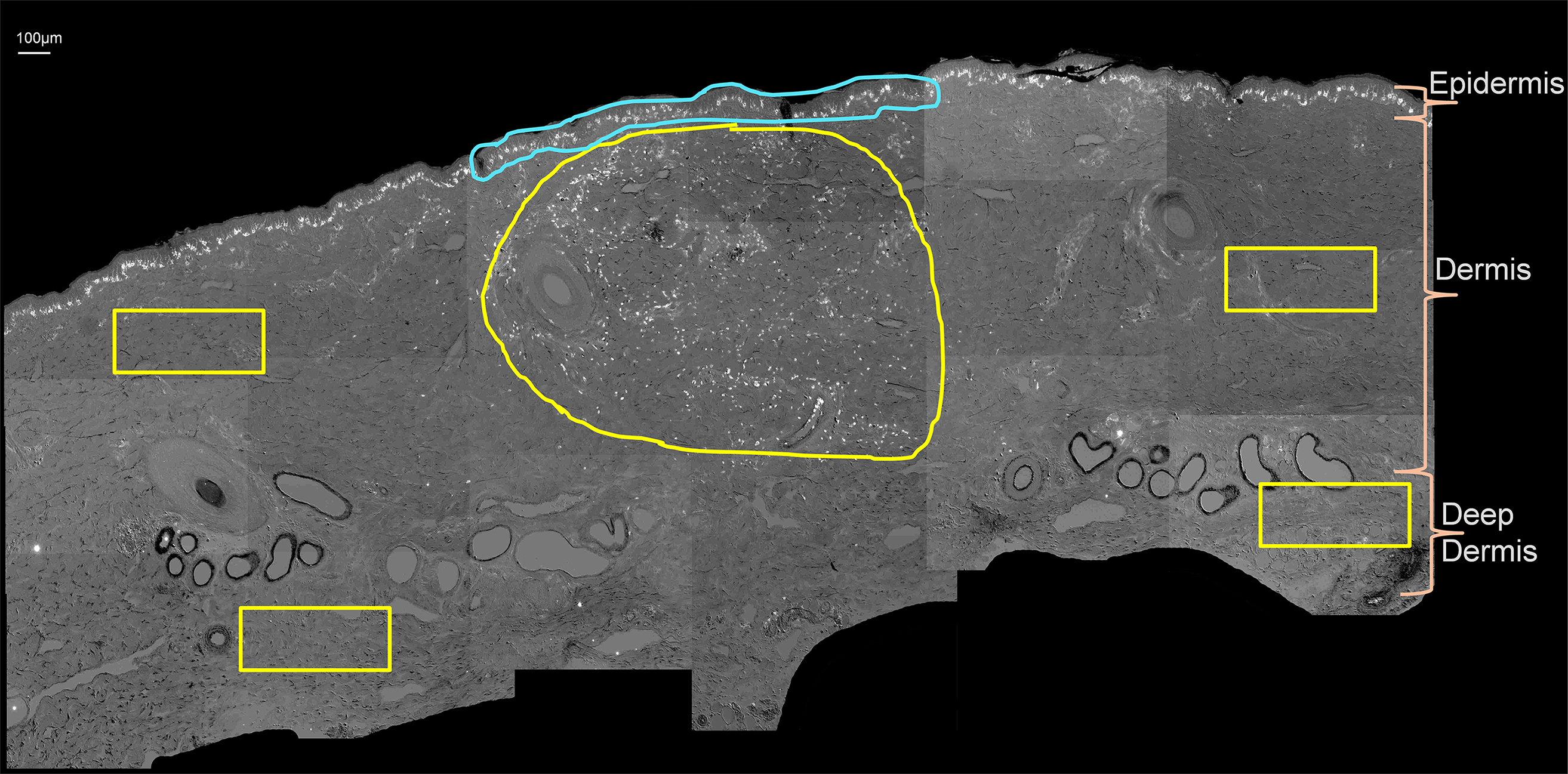

Supplement: S1 Fig — Within the epidermis, the transect for the Langerhans cell count is delineated in blue. Within the dermis and deep dermis, the macrophages were counted inside the areas delineated in yellow. The circular area corresponds to the area of the tick bite or inoculation point. The yellow rectangles are outside the area where the tissue was disrupted. In the opposite ear, macrophages were only counted in the yellow rectangles as there was no tissue disruption and the transect extended throughout the biopsy. (TIF) [file pone.0147869.s001.tif]

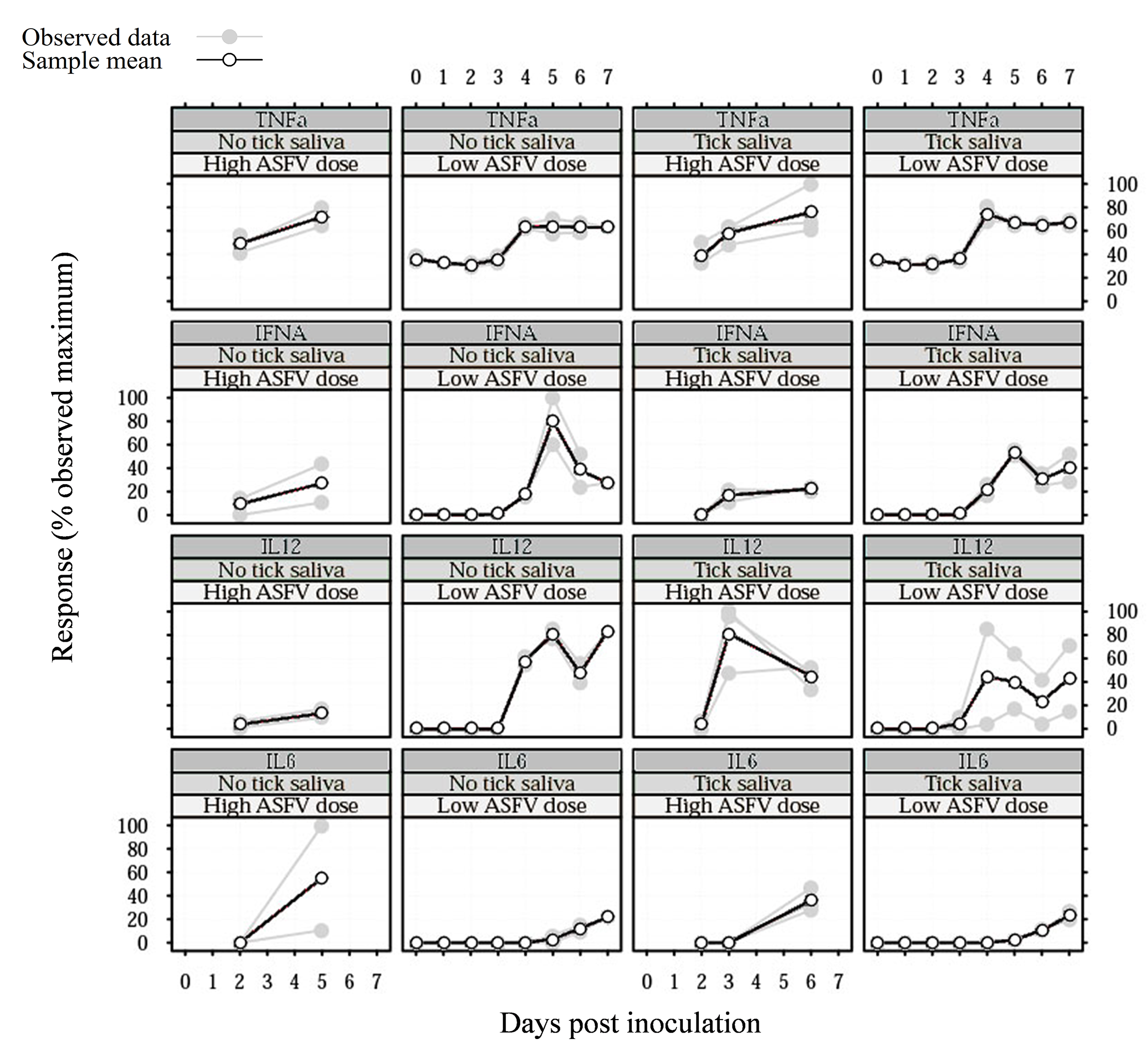

Supplement: S2 Fig — For each cytokine, whatever the pig group and day post infection, the results were transformed into a percentage of serum concentration, with the maximal observed concentration considered to have 100% activity. (TIF) [file pone.0147869.s002.tif]

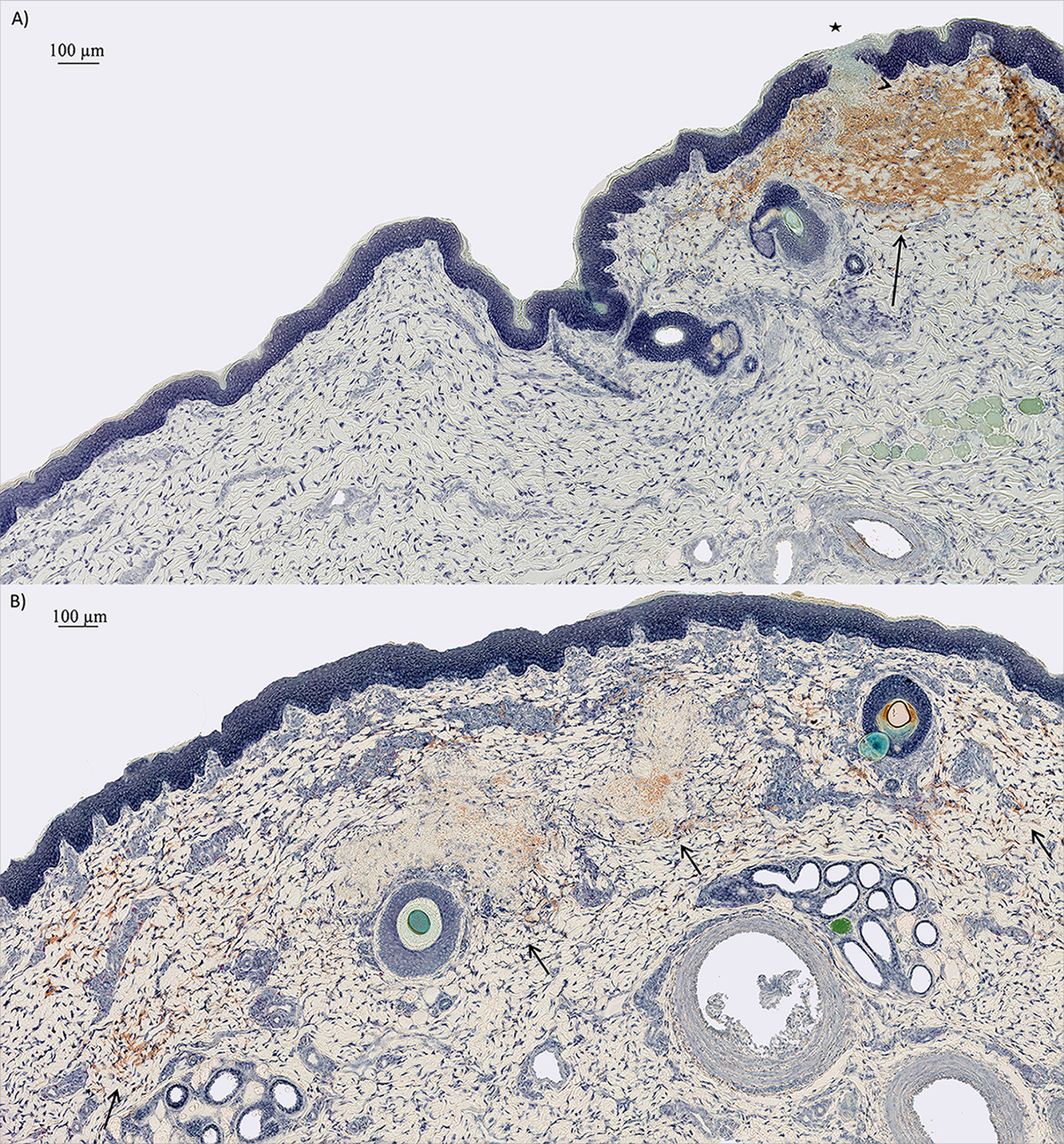

Supplement: S3 Fig — (A) Tick bite at 1 h pi, black star showing the tick bite, an arrow indicating the haemorrhagic area. (B) Tick bite 48 h pi with arrows showing extensive haemorrhagic area and dermis lesions. Staining: Hemacolor kit (Merck Millipore, Darmstadt, Germany) (TIF) [file pone.0147869.s003.tif]

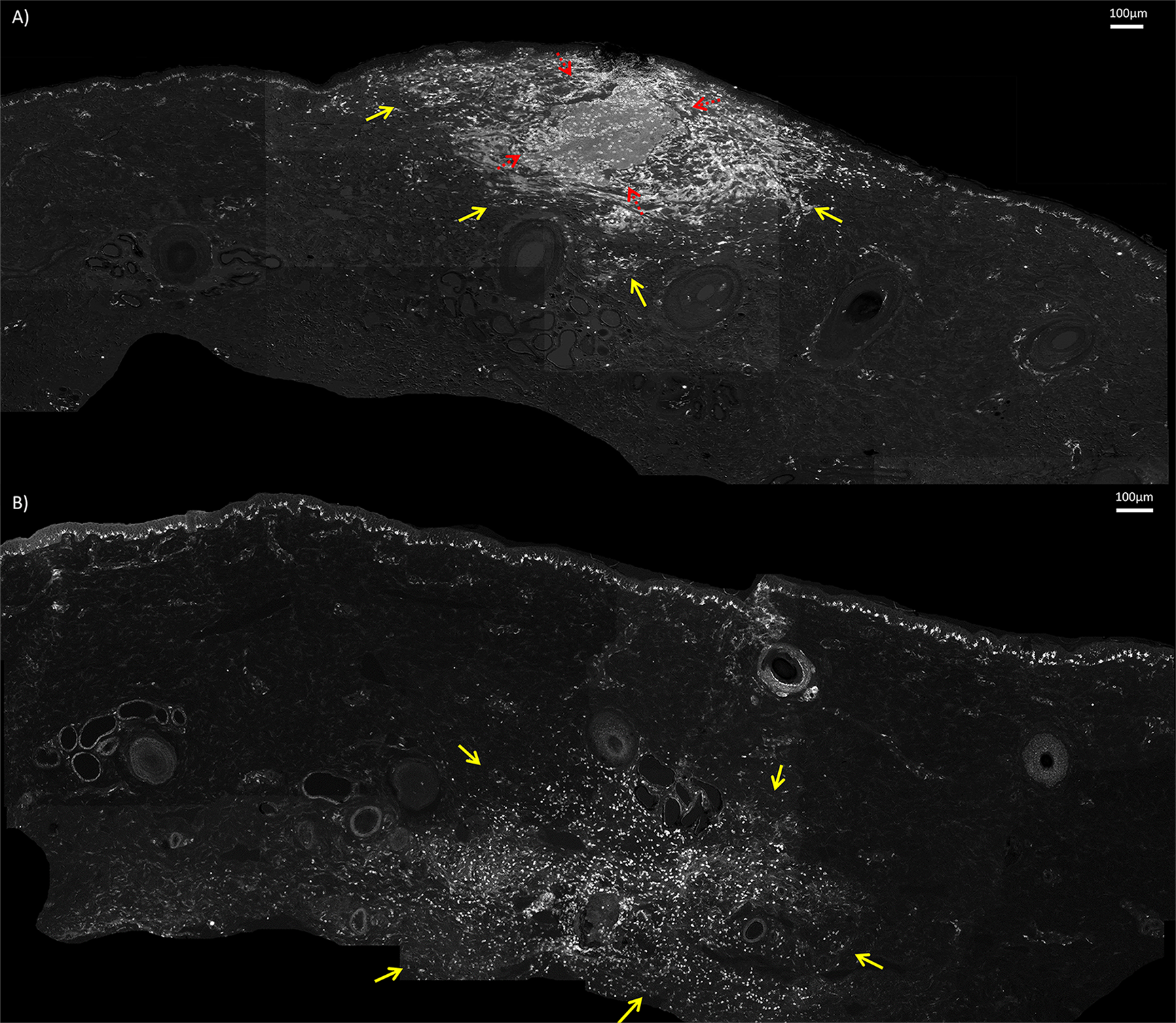

Supplement: S4 Fig — (A) Tick bite. (B) Intradermal inoculation. Two types of tissue lesion were observed. The first was characterized by an area delimited by tissue disruption, collagen disruption and more abundant SWC3/CD172 cell labelling than in healthy tissue (yellow arrows). The second was sometimes observed inside the first, consisting of more intensive damage with more abundant haemorrhage and SWC3/CD172 cell labelling (red arrows). Most of the TICK biopsies presented both areas. However, statistical model analyses were only performed on the disrupted area indicated in the photo by the yellow arrows. Biopsies in the samples with tick bites were performed to a depth of 550,0 μm (n = 14 biopsies on 7 pigs), unlike the inoculated biopsies which were performed to a depth of 1052.8 ± 511.4 μm (n = 23 biopsies on 14 pigs). (TIF) [file pone.0147869.s004.tif]

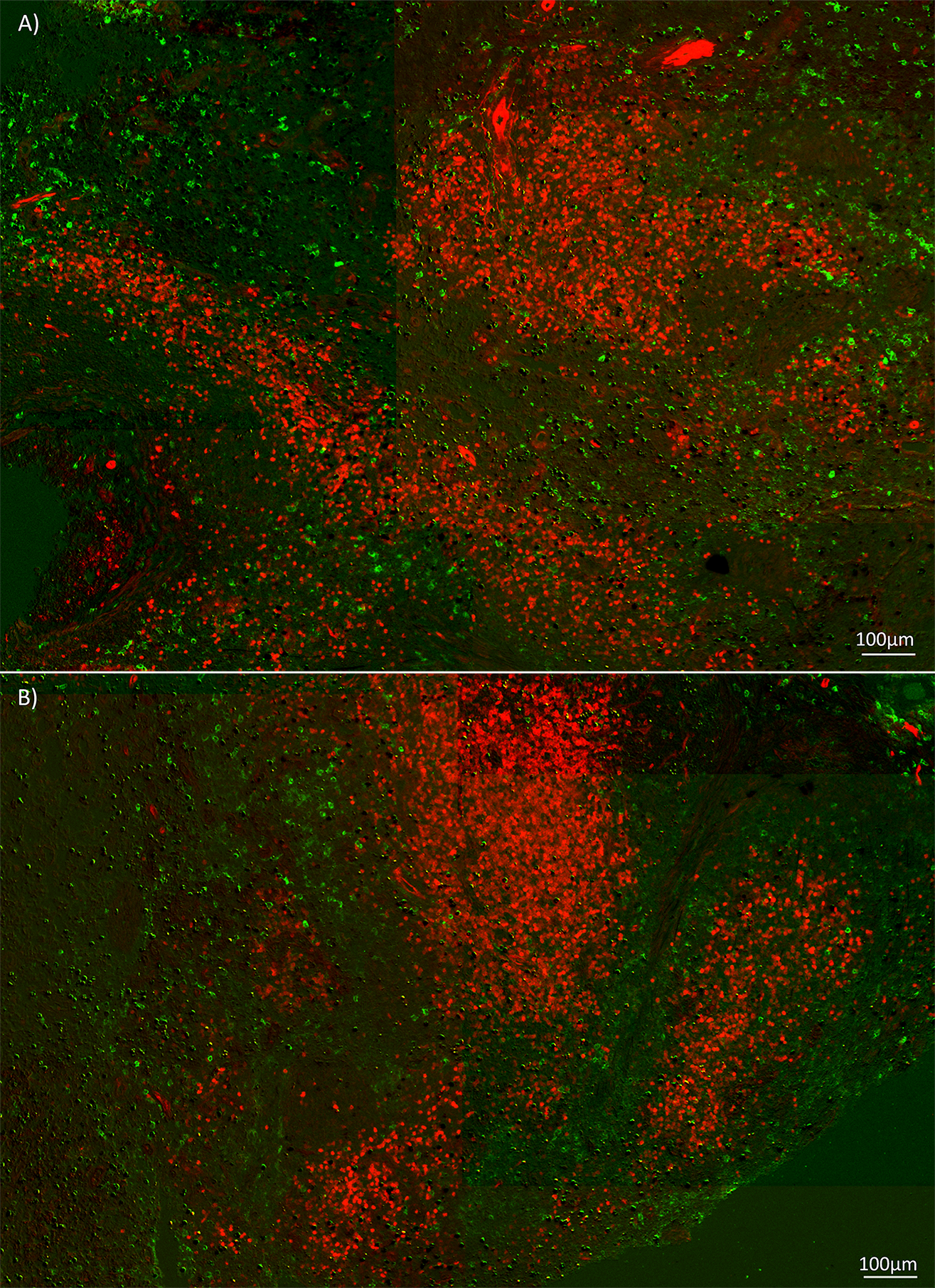

Supplement: S5 Fig — (A) Parotid lymph node from inoculation side for pigs receiving a high ASFV dose and tick salivary gland extract. (B) Parotid lymph node from inoculation side for pigs receiving a high ASFV dose alone. Merging of histological slice labelled in red with S100-Ab (IgG1, interdigitating dendritic cells, Clone SH-B1, Sigma) [18] and in green with SWC3-Ab. (TIF) [file pone.0147869.s005.tif]
